# Ethical aspects in tissue research: thematic analysis of ethical statements to the research ethics committee

**DOI:** 10.1186/1472-6939-13-20

**Published:** 2012-08-08

**Authors:** Arja Halkoaho, Anna-Maija Pietilä, Mari Vesalainen, Kirsi Vähäkangas

**Affiliations:** 1 Faculty of Health Sciences, University of Eastern Finland, Research Unit/Research Ethics Committee, University Hospital of Kuopio, P.O Box 1777, 70210 , Kuopio, Finland; 2 Faculty of Health Sciences, University of Eastern Finland, Social and Health Care Services, Kuopio, Finland; 3 Faculty of Health Sciences, University of Eastern Finland, Kuopio, Finland

**Keywords:** Thematic content analysis, Ethical views of scientists, Application to ethics committee, Informed consent, Recruitment

## Abstract

**Background:**

Many studies have been published about ethics committees and the clarifications requested about the submitted applications. In Finland, ethics committees require a separate statement on ethical aspects of the research in applications to the ethics committee. However, little is known about how researchers consider the ethical aspects of their own studies.

**Methods:**

The data were collected from all the applications received by the official regional ethics committee in the Hospital District of Northern Savo during 2004–2009 (n = 688). These included a total of 56 studies involving research on tissue other than blood. The statements by the researchers about the ethics about their own research in these applications were analyzed by thematic content analysis under the following themes: recruitment, informed consent, risks and benefits, confidentiality and societal meaning.

**Results:**

The researchers tended to describe recruitment and informed consent process very briefly. Usually these descriptions simply stated who the recruiter was and that written consent would be required. There was little information provided on the recruitment situation and on how the study recruiters would be informed. Although most of the studies were clinical, the possibility was hardly ever discussed that patients could fail to distinguish between care and research.

**Conclusion:**

The written guidelines, available on the webpages of the ethics committee, do not seem to be enough to help researchers achieve this goal. In addition to detailed guidelines for researchers, investigators need to be taught to appreciate the ethical aspects in their own studies.

## Background

Incidents of questionable practices in human biomedical research in the history have triggered the need for international regulations e.g. the Nuremberg Code in 1947 and later the Declaration of Helsinki in 1964. These define the ethical principles of medical research involving human subjects. In the past decades, a number of regulations about medical research have been issued at both the international and national levels [1]. According to the Declaration of Helsinki, a pre-evaluation of the research plan should be performed by an independent research ethics committee [1,2]. According to the current Finnish legislation, five health care districts have official research ethics committees that evaluate all biomedical studies on humans and human tissues from an ethical and legal perspective [3].

Studies investigating the process of ethical review have revealed that most of the clarifications requested from the researchers by the committees are concerned with the informed consent process [4,5]. There may be other causes for clarification requests e.g. failure to comply with correct procedures, missing information and discrepancies [6]. In studies on the evaluation process itself, the critical aspects have included the evaluation time [7], a lack of uniformity in application forms and variations in the approval procedures adopted by different committees within the same country [8,9].

Whitney and coworkers [10] focused on the opinions of researchers about the functions of the Independent Review Board (IRB) system. Their results indicated that some scientists consider the IRB system to be cumbersome by requiring completion of incomprehensible consent forms that focus too much on details and which seem to be intended to protect the institution rather than the research subjects [10]. In addition, failure of assessing risks and benefits, scientific validity and handling of consent procedures has been criticized by Paul [11]. Taylor and coworkers suggested that one possibility to speed up the evaluation process would be to invite the principle investigator to attend the review meetings [12]. Good communication between the scientists and the ethics committee is critical if one wishes to achieve a thorough consideration of all of the ethical aspects of research. Prior attention should be paid beforehand to ethical aspects if one wishes to guarantee the protection of research participants as well as making sure that there are no misunderstandings about the goals of the study [12-14]. Education and training are also considered important for IRB members [15].

Several ethical aspects, especially those involving tissue research, have been highlighted in the literature. Legislation about the use of human tissue in scientific research is regarded as important [16,17] and not unexpectedly, informed consent and autonomy in all human research are listed as central issues [17-20]. Today, the challenges of data protection and good research governance are emerging with the development of large tissue and gene banks [17]. Another example of a specific type of tissue research is human placental perfusion studies which have recently been under scrutiny from an ethical point of view [20-23].

The purpose of this study was to evaluate the ethical statements of principal investigators. Such statements are required by the official Research Ethics Committee of the Hospital district of Northern Savo. We limited this study to tissue research, excluding studies involving only blood samples.

## Methods

### Data collection

The data were collected from applications received by the Official Regional Ethics Committee of the Hospital District of Northern Savo during 2004–2009. In the years 2004–2005, the application form had a space for ethical considerations. However, it was possible to include a separate statement sheet as an attachment. At that time, there were no explicit guidelines concerning the structure of the ethics statement. From the beginning of 2006, a separate statement sheet on research ethics was required to be submitted as an attachment to the application form. This was intended to include the following topics: justification for the research (including societal meaning), informed concent process, voluntariness, and recruitment process, whether any vulnerable groups (e.g. children or pregnant women) would be included, confidentiality, and evaluation of risks and benefits.

The data were manually collected by two authors (AH, MV) from all the applications sent to the ethics committee during the defined years (n = 688). Although the material was collected by two researchers, the multidisciplinary research group discussed the selected studies. Studies planning to include only a blood sample, or tissue from a deceased person were excluded. Altogether 56 cases involving tissue research were available. Ethics committee guidelines listing the required documents are available in the Internet (http://www.kuh.fi; Table 1).

**Table 1:** Required documents in tissue research by the official Research Ethics Committee of Hospital District of Northern Savo

**Documents required by the research ethics committee**	**Required main contents**	**Justification for the need**	**Relevant legislation**
**Application form**	General detailed information of the study (e.g. scientists, place of study)	2004-2005 included the ethics statement by principal investigator	Not based on law, administrative order
**Study Plan**	All scientific details of the study	Scientifically essential	Medical Research Act 488/1999
**Information form**	All information about the study for the potential participants	To check that it is understandable by lay people	Medical Research Act 488/1999, Act of Medical Use of Human Organs and Tissues 101/2001
**Consent form**	Clarification for what the participants are actually consenting	Signed permission of the participant to be involved in the study	Medical Research Act 488/1999
**Separate statement about research ethics by the principal investigator**	Ethical justification of the study; how ethical principles will be taken into on account in practice	To ensure legally and ethically defensible practices	Not based on law; practice of the research ethics committee
**Document explaining the registry of personal data files**	Included personal data, access of data, storage and disposal, responsible person	To ensure personal data protection	Personal Data Act 523/1999
**Other documents e.g. questionnaire, letters, advertisements**	Type and contents depend on the particular study	To check understandability, legality and discretion	Medical Research Act 488/1999

This research project received an administrative approval by the participating hospital. According to the Finnish law [3] this type of study does not need the approval from an official research ethics committee. The data was stored in a locked place and coded for analysis.

### Data analysis

Within the documents, the data were analyzed both qualitatively and quantitatively according to five themes: recruitment, informed consent, risk and benefits, confidentiality and societal meaning of the research. The statements were read by AH and meaningful concepts and information were grouped under the selected themes and a thematic content analysis was performed [24,25]. If a theme was addressed even in one sentence, this was included as a statement within the theme. Although the material was analysed by one researcher, the multidisciplinary research group discussed the results at all stages of the analysis. The research group also worked together in reviewing the conceptualisation process and the selected concepts by confirming the validity of the study [24,25]. The content of the themes (Table 2) was formulated according to the literature [2] and previous studies of our research group [20,22,23] as well as taking into consideration the instructions of the ethics committee.

**Table 2:** **Preconceived aspects from the literature within the themes**[2,20,22,23]

**The main aspects**	**Contents sought out**
**• Recruitment**	• Situation at the time of the research
	• The process of decision about participation
	• How the information was planned to be given to recruiters
	• Possible vulnerable groups
**• Informed consent and voluntariness**	• How the information was planned to be given to invited participants
	• Comprehension of written and oral information
	• Voluntariness
	• Appreciation of voluntariness
	• Interaction between the recruiter and participants
**• Risks and benefits of the research for participants**	• Meaning, significance and stressfulness of the research for the participants
	• Purely research or within clinical care
**• Handling and confidentiality of personal data**	• Confidentiality, concerns for preserving privacy
**• Views about societal meaning**	• General justification of research
	• Putative benefits for future

## Results

The selected cases represented different types of tissues, e.g. cancerous tissue, placental tissue, fatty tissue, brain and neurons, gynecological tissues and samples from the gastrointestinal tract. All of the application forms from the years 2004–2005 contained text about ethical aspects of the research project. In addition, a separate statement of research ethics was included in 8/24 of these statements. In 2006–2009, after the new guidelines were implemented by the ethics committee, all of the researchers submitted a separate statement as required in the guidelines.

There were differences in the contents of the statements both when comparing the years 2004–2005 with 2006–2009, and between the statements within these groups. In general, the statements varied from a simple statement that the Declaration of Helsinki was taken into account, to detailed descriptions of many aspects of research ethics (Table 3).

**Table 3:** Presence of ethical themes in the analyzed statements

**Theme**	**Years 2004–2005 (n = 24)**	**Years 2006–2009 (n = 32)**	**Total (n = 56)**
	**No. (% of total)**	**No. (% of total)**	**No. (% of total)**
**Recruitment**	4 (16.7)	27 (84.4)	31 (55.4)
**Informed consent**	9 (37.5)	24 (75.0)	33 (58.9)
**Risks and benefits**	16 (66.7)	29 (90.6)	45 (80.4)
**Confidentiality**	10 (41.7)	26 (81.3)	36 (64.3)
**Societal meaning**	6 (25.0)	13 (40.6)	19 (33.9)

### Recruitment and informed consent

The recruitment situation was described in more than a half (31/56) of all the studied statements (Table 3). On the other hand, recruitment was addressed only in four of the statements from the years 2004–2005 and generally only the study population was defined (e.g. diabetics). In the statements from the years 2006–2009, the recruitment process was explained in 27/32 of the statements and more information was given; e.g. about the planned recruiters, recruitment situation and how the recruiters would be informed. In 5/56 statements, the researchers had actually considered that the recruitment situation was sensitive and this fact was described in detail. These five statements also included a plan to include information about the life-situation of the research participants during the recruitment. The rest of the statements mentioned very briefly the plan for recruitment and the informed consent process: who would be doing the recruitment and how the information was planned to be given to the participants. In most cases (46/56), the recruitment was planned to take place during clinical treatment e.g. surgery or birth. The planned recruiters varied (nurses, physicians or researchers).

The informed consent process was described in 33 of the 56 statements. In general, the most common description of the informed consent process stated that there would be a request for written informed consent and the possibility to refuse participation. Voluntariness and the possibility to discontinue the research were described in 6/56 statements. A total of 8/56 statements mentioned that time would be given for the invited participants to consider their decision. However, in most cases, the actual time allocated for a decision was not defined.

### Handling and confidentiality of personal information

Handling and confidentiality of personal information were described in 36/56 of the statements. Coding was correctly explained in 29/56 and anonymization in 5/56. In these cases, coding and anonymization were defined in a way that allowed the reader to understand what the author meant by these terms. However, in 3/56 statements, the meaning of anonymity (no possibility to link data to a person) and coding was confused (as an example *“samples will be anonymized and coded”*)*.* In 28/56 cases, there was information provided about which individual would have the code key and in 18/56 it was explained how the data would be stored.

### Risks and benefits, and societal meaning of the research

Considerations about risks and benefits were written in 80.4 % of all the statements. In most of these cases, it was stated that the tissue would be removed during a clinical procedure. The authors often stated that therefore no risk for research participants would be envisioned, and/or mentioned that the tissue was regarded as waste. Additional samples (a separate tissue sample, other samples, e.g. blood) were, however, explained in detail and considered as an extra risk. On the other hand, in all cases, the difference between research and care was left unexplained. Individual research participants in 11/56 cases were informed that they would not gain any direct benefit from tissue donation. However, the, scientists did state that benefits would be conferred through certain other factors, for instance by obtaining results of blood tests or through health education. In some studies, this type of information was planned to be given to the potential participants in an information leaflet.

In their consideration of ethical aspects, four researchers mentioned the possibility of unexpected results and how the research participants in such a case would be given the best possible care. None of the statements contained any consideration under the term “societal meaning”. However, the following aspects mentioned by the scientists could be regarded as belonging to this category: 11/56 mentioned that one benefit would be the development of new methods, and 13/56 referred to benefits for public health care in general (e.g. clarification of disease mechanisms providing possibilites for preventive actions and better care).

## Discussion

Although there have been publications on the ethical aspects of tissue research, as far as we are aware, none have so far handled the ethical statements of researchers. The Research Ethics committee of Hospital District of Northern Savo expects that a separate statement sheet should be submitted to the research ethics committee. One interesting and important point emerging from our results was the insufficient handling of the key ethical aspects in many of the applications for the research ethics committee. On the other hand, only the official forms and separate statements about ethical aspects were studied and it is possible that the research plan in some cases included some of this information. It was evident that, in general, the statements were less structured in 2004–2005 than in 2006–2009, when the ethics committee had already formulated the requirements for the ethical statements and their content. Thus, in our case, the formal requirement and advice by the ethics committee had a positive effect on the quality of the written statements. In the study by Antes and coworkers [26] it was disturbing that in some cases the reasoning strategies, such as awareness of the situation and consideration of personal motivations, improved but at the same time there was a decline in seeking help and considering others' perspectives. Mentoring in research ethics is a two-way road also with the possibility to both increase and decrease ethically good conduct [27]. One postulated way to improve ethics in biomedical research is to conduct an ethics consultation with a bioethicist [28]. However, at present it is still unclear when, how and by whom research ethics instruction would be most beneficial. In addition, there needs to be a systematic study of how such measures could affect the actual ethical conduct of researchers in addition to recognition and formulation of ethical aspects and their handling in written statements.

According to the general consensus, ethical considerations should include details of the recruitment and informed consent process with the following elements: competence, voluntariness, understanding and consent [1,2]. Still, only a few of the studied statements discussed how competence and understanding would be ensured. Voluntariness was usually mentioned very briefly in one sentence. In our earlier studies [20,22,23] in accordance with the literature [29], we have found the importance of considering the actual situation when the signature is being gained from the participant. In this study, the ethical aspects about obtaining the signature for the consent form were almost forgotten by the researchers, at least according to their statements. Very few statements mentioned how much time would be available for the participant to make a decision. However, the time given has been considered an important detail by many authors [e.g. [13,30,31].

A major issue is how to educate the recruiters when they are not part of the research group: how to inform nurses/doctors about the research project in such a way that they can provide relevant information to the participants [22,23]. This aspect was missing in most of the statements, when the recruiter came from outside the research group. Furthermore, there was a failure to explain the difference between patient care and research to the patient. This so-called therapeutic misconception, which means that the participant thinks that the study is part of some clinical treatment, is a common situation in practice [32,33]. To obtain a genuine informed consent, these aspects should be discussed when planning the research. Furthermore, in our previous studies [20,22,23] and other authors [34,35] have observed that even if planning is done carefully and information is given to the recruiters, the recruitment in practice can be very challenging.

The guidelines of the Ethics Committee of the Hospital District of Northern Savo instruct researchers to consider the justification for the research in their ethical statements (Table 1). Furthermore, the Declaration of Helsinki, as well as other regulations, requires that research should be based on earlier literature and this should be demonstrated in the research plan [1]. One of the most important justifications for biomedical research is its usefulness for patients and patient care in the form of applicable knowledge for diagnostic, therapeutic and prognostic purposes. Nonetheless, the studied statements provided little information on the societal meaning of the study. Similarly to confidentiality, societal justification is a topic that may not be perceived as an ethical aspect, and most likely it has been described in the research plan.

It is unfortunate that the terminology in the literature is confusing, for instance, the definitions of anonymity and coding leave room for misunderstanding [13]. It is clearly challenging to make the studies understandable to lay people and such poorly defined details confuse both researchers and the participants. Thus, the scientific community should attempt to clarify the scientific terminology, which actually may be regarded also as an ethical requirement in human studies.

In future tissue research, it would be important to study whether it makes a difference to the participants if the tissue is removed simply for research purposes or if it is leftover tissue from routine operations. In addition, it would also be interesting to determine how people perceive the difference between the situation of healthy volunteers asked to donate to tissue banks compared to the views of actual patients asked to donate left-over tissue. Considering that this study was carried out in one center only, we do not know the generalizability of our results and comparison of investigations from different centers would be helpful in further studies.

## Conclusion

In conclusion, the justification of the research from an ethical point of view was missing from the majority of the statements. It is noteworthy that even after the requirement by the Ethics Committee for an ethical consideration became mandatory, a significant percentage of applications lacked considerations of recruitment, informed consent and confidentiality. In view of the rapid developments occurring in biomedicine, it is clear that there is a need for continuing education in research ethics. In addition, a dialogue between the scientists and society would provide a better foundation for scientists to take the societal aspects of their research into account.

## Competing interests

The authors declared that they have no competing interest.

## Authors’ contributions

KV, AMP and AH designed the study. AH and MV collected the data. AH analyzed the data and discussed the analysis with KV and AMP. KV, AMP and AH wrote the paper. All authors read, commented and accepted the final version.

## Funding

This study has financially supported by Kuopio University Hospital EVO-funding.

## Pre-publication history

The pre-publication history for this paper can be accessed here:

http://www.biomedcentral.com/1472-6939/13/20/prepub
